# Changes at the Pharmaceutical Society

**Published:** 1918-06-22

**Authors:** 


### Changes at the Pharmaceutical Society.

For the first time in the seventy-seven years of the Pharmaceutical Society's history a Scotsman has been elected as president of that body, Mr. W. L. Currie, of Glasgow succeeding Mr. Edmund White, who did not seek re-election. There is also a new treasurer—Mr. F. Bilson, of Bournemouth; the post of secretary and registrar is temporarily vacant through the resignation of Mr. W. J. U. Woolcock. It was generally expected that Mr. W. S. Glyn-Jones, M.P. for Stepney, would have been appointed secretary and registrar at the June Council Meeting, but at the last moment a hitch occurred, and no decision was arrived at.

Mr. Glyn-Jones has been the Pharmaceutical Society's Parliamentary Secretary for a number of years, and has everything at his finger tips; it is probable that his appointment has merely been postponed for a month. It cannot be said that the recent changes bring the Society into closer touch with hospital pharmacy; both the late president, Mr. White, and the late secretary, Mr. Woolcock, had a wide knowledge of institutional dispensing gained by actual experience, the one at St. Thomas's Hospital, and the other at University College Hospital.

So far as we know neither Mr. Currie nor Mr. Glyn-Jones has had any large experience in hospital work, at any rate, for many years past, although no doubt they are fully informed as to the work of hospital dispensers and in sympathy with them.

